# Assessment of Glutathione Peroxidase-1 (GPX1) Gene Expression as a Specific Diagnostic and Prognostic Biomarker in Malignant Pleural Mesothelioma

**DOI:** 10.3390/diagnostics11122285

**Published:** 2021-12-07

**Authors:** Amal F. Gharib, Muhammad Alaa Eldeen, Amany Salah Khalifa, Wael H. Elsawy, Emad Mohamed Eed, Ahmad El Askary, Refaat A. Eid, Mohamed A. Soltan, Nermin Raafat

**Affiliations:** 1 Department of Clinical Laboratory Sciences, College of Applied Medical Sciences, Taif University, P.O. Box 11099, Taif 21944, Saudi Arabia; amgharib@tu.edu.sa (A.F.G.); e.eed@tu.edu.sa (E.M.E.); a.elaskary@tu.edu.sa (A.E.A.); 2 Cell Biology, Histology, Genetics Division, Zoology Department, Faculty of Science, Zagazig University, Alsharquia 44519, Egypt; 3 Department of Clinical Pathology, Faculty of Medicine, Menoufia University, Menoufia 32511, Egypt; dramanisalah@yahoo.com; 4 Department of Clinical Oncology, Faculty of Medicine, Zagazig University, Zagazig 44519, Egypt; whelsawy@gmail.com; 5 Department of Pathology, College of Medicine, King Khalid University, Abha 12573, Saudi Arabia; refaat_eid@yahoo.com; 6 Department of Microbiology and Immunology, Faculty of Pharmacy, Sinai University, Ismailia 41611, Egypt; mohamed.mohamed@su.edu.eg; 7 Department of Medical Biochemistry, Faculty of Medicine, Zagazig University, Zagazig 44519, Egypt; nerminraafat@gmail.com

**Keywords:** malignant pleural mesothelioma, GPX1, gene expression, diagnostic biomarker

## Abstract

Malignant pleural mesothelioma (MPM) is a malignant tumor of the mesothelial lining of the thorax. It has been related to frequent exposure to asbestos. Diagnosis of malignant pleural mesothelioma is considered a criticizing problem for clinicians. Early diagnosis and sufficient surgical excision of MPM are considered the cornerstone success factors for the management of early MPM. Glutathione peroxidase-1 (GPX1) is an intracellular protein found to be extensively distributed in all cells, and it belongs to the GPX group. In the current study, we included ninety-eight patients with MPM that underwent surgery at the Zagazig University Hospital in Egypt. We assessed GPX1 gene expression level as it was thought to be related to pathogenicity of cancer in a variety of malignant tumors. We observed a significant elevation in GPX1-mRNA levels in MPM relative to the nearby normal pleural tissues. It was found to be of important diagnostic specificity in the differentiation of MPM from normal tissues. Moreover, we studied the survival of patients in correlation to the GPX1 expression levels and we reported that median overall survival was about 16 months in patients with high GPX1 expression levels, while it was found to be about 40 months in low GPX1 levels.

## 1. Introduction

Malignant pleural mesothelioma (MPM) is a neoplasm caused by asbestos exposure and originates in the mesothelial lining of the thoracic cavity [1]. MPM is a fatal tumor; its prognosis is marginally impacted by the current cancer treatments [2]. It represents a critical problem for clinicians in its diagnosis and treatment. MPM is usually presented in late stages with a median survival of less than one year [3]. Early diagnosis and adequate treatment prove to remain the predominant factors in the treatment of early MPM. However, until now, there are no dependent and accurate biomarkers for its detection and diagnosis; at the time of diagnosis, nearly all cases have advanced or metastatic tumors [4].

The glutathione peroxidase (GPX) is one of the groups of the phylogenetic trees associated enzymes. GPX is involved in numerous vital biological processes. GPX induces H_2_O_2_ reduction and glutathione (GSH) oxidation that can be reduced again by GPX reductase via NADPH [5]. GPX1, a widely released component of the GPX group, is an intracellular protein extensively distributed in all cells [6]. The expression of GPX1 is directly linked with malignant transformation and tumor spread. It has been observed in many types of malignancies [7].

Interestingly, literature also showed a significant impact of GPX1 on the inhibition of malignant transformation by reducing the oxidative DNA mutations during the initiation of the transformation process [8]. One study reported that GPX1 overexpression inhibited the proliferation of malignant pancreatic cell lines [9]. Baliga et al. observed that GPX1 knockdown in prostate cancer cells enhanced the radiotherapy effect by increasing the formation of micronuclei [10]. Higher GPX1 expression, on the other hand, was associated with substantial penetration of the blood vessels and low survival in hepatocellular carcinoma and oral squamous cell carcinoma [11,12].

Considering the data described earlier, GPX1 may have a protective or injurious biological function depending on the type of malignant tumor. The role of GPX1 in malignancy remains relatively unclear, requiring further research, especially on the relationship between GPX1 and the clinical outcome. In MPM, the role of GPX1 and its function was not previously studied; therefore, we conducted this research to evaluate its role and its diagnostic and prognostic capability in MPM.

## 2. Materials and Methods

Ninety-eight patients with locally or early advanced MPM participated in this study. Most of patients were surgically resectable. Patients did not receive neoadjuvant therapy before surgery. They were surgically operated at the Zagazig University Hospital, Egypt, during the period from March 2014 to December 2020. Informed consent was obtained from all included patients and approval of the study was obtained from the ethical committees of the Zagazig University. All patients were clinically staged according to the eighth edition manual of the American Joint Committee of Cancer (AJCC) [13]. The performance status of all patients was assessed using the Zubord Scale [14].

### 2.1. Tissue Specimens

Tissue specimens were obtained from the frozen specimens collected from the malignant and the adjacent normal pleural tissues (ANPT) excised during surgery.

### 2.2. RNA Isolation

Total RNA was extracted from 50–100 mg of grounded frozen tissue specimens by the miRNeasy Mini Kit (QIAGEN, Hilden, Germany) in compliance with the supplier’s instructions. Each sample was put in 600 μL RNA lysis buffer (Promega, Madison, Wisconsin) until complete digestion of the tissues occurred. The final extracted RNA volume was 40 µL.

### 2.3. Synthesis of Complementary DNA (cDNA)

The extracted RNA was reverse transcribed to synthesize cDNA by iNtRON Biotechnology, cat No 25011, Korea, in compliance with their manual.

### 2.4. GPX1 Gene Expression Analysis by RT-qPCR

The quantitative real-time polymerase chain reaction (RT-qPCR) was carried out by SYBR Green PCR Master Mix (Perfect Real Time; TaKaRa Biotechnology, Shiga, Japan) using StratageneMx3005P-qPCR system. The amplification reaction was performed using 20 μL of a mixture composed of 5 μL cDNA, 10 μL SYBR Green master mix, and 100 pmol/ul of the primers. The β-actin gene was utilized for internal control. We used the following primers: GPX1 forward primer (5′-3′): GTG CTC GGC TTC CCG TGC AAC and reverse primer (5′-3′): CTC GAA GAG CAT GAA GTT GGG C; β-actin forward primer (5′-3′): CAC CAT TGG CAA TGA GCG GTT C; and the reverse primer (5′-3′): AGG TCT TTG CGG ATG TCC ACG T.

All tests were utilized and measured in duplicate and normalized to the levels of β-actin. The GPX1gene expression fold change was quantitated by the 2^−ΔΔCt^ method [15].

### 2.5. Statistical Analysis

Quantitative data were expressed as the mean ± standard deviation and compared using the Student’s (*t*-test) and one-way analysis of variance (ANOVA). *p* < 0.05 was deemed significant. The capability of GPX1 gene expression to differentiate between malignant and healthy tissue was analyzed by plotting receiver operating characteristics curve (ROC), which relates the true positive (sensitivity) to the false positive (specificity), and by computing area under the curve (AUC). Survival was evaluated by the Kaplan–Meier curve. All statistical estimates were carried out by SPSS version 23.0 (SPSS Inc., Chicago, IL, USA).

## 3. Results

### 3.1. GPX1-mRNA Levels in MPM and Their ANPT 

**Figure 1:**
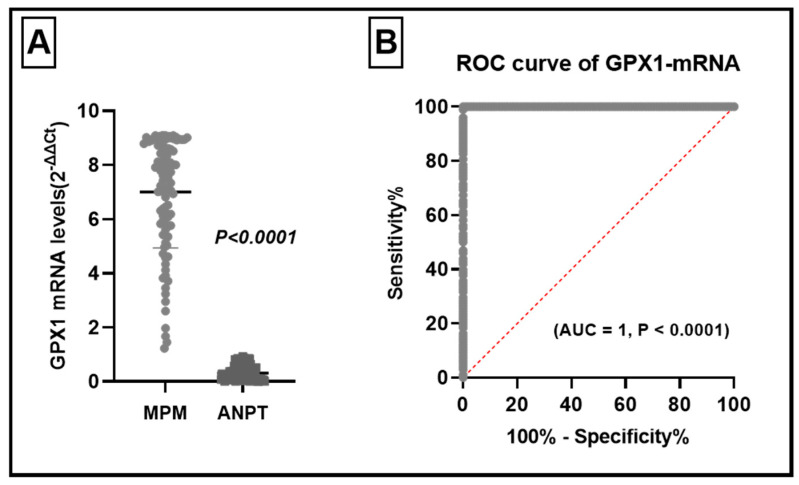
GPX1 levels in malignant pleural mesothelioma (MPM) and their adjacent normal pleural tissues (ANPT). (**A**) GPX1-mRNA levels were significantly higher in MPM (t = 31.87, *p* < 0.0001). (**B**) ROC curve analysis of levels of GPX1-mRNA in MPM and ANPT, with high ability to distinguish MPM (AUC = 1.95%, CI: 1.000 to 1.000, *p* < 0.0001).

The GPX1-mRNA levels were significantly elevated in MPM relative to ANPT; in MPM it ranged between 1.2 to 9.09 (2^−ΔΔCt^) with a mean value of 7.001, while in ANPT it ranged between 0.005 and 0.92 with a mean value 0.31. This variation was statistically significant (Student’s *t*-test, t = 31.87, *p* < 0.0001) (Figure 1A). 

To find out if the high levels of GPX1-mRNA have a diagnostic specificity for MPM, we plotted the receiver operating characteristic (ROC) curve. The ROC curve study revealed that GPX1-mRNA could accurately differentiate MPM tissues from normal pleural tissues with an area under the curve (AUC) of 1.95% (CI: 1.000 to 1.000, *p* < 0.0001) (Figure 1B).

### 3.2. Clinical Significance of Elevated GPX1-mRNA Levels in MPM

We probed the elevated GPX1 levels and different prognostic, clinical, and pathological features. Elevation of GPX1 levels were noted in older patients >55 years (*p* < 0.0001), female patients (*p* < 0.0001), non-epithelioid histopathological type (*p* < 0.0001), high grade tumors (*p* < 0.0001), large tumors (*p* < 0.0001), lymph node involvement (*p* < 0.0001), advanced stage II clinical stage (*p* < 0.0001), patients with anemia (*p* < 0.0001), patients with increased platelet counts, white blood cells and LDH levels (*p* < 0.0001). The bad performance status (PS) of the patient was significantly related to the elevation of GPX1-mRNA levels (*p* < 0.0001). Unrespectability of the primary tumors were also found to be associated with high GPX1-mRNA levels (*p* < 0.0001) (Table 1). Elevated GPX1-mRNA levels were found to be linked to adverse prognostic and clinical features.

### 3.3. The Role of Elevated GPX1- mRNA in the Diagnosis of MPM

**Figure 2:**
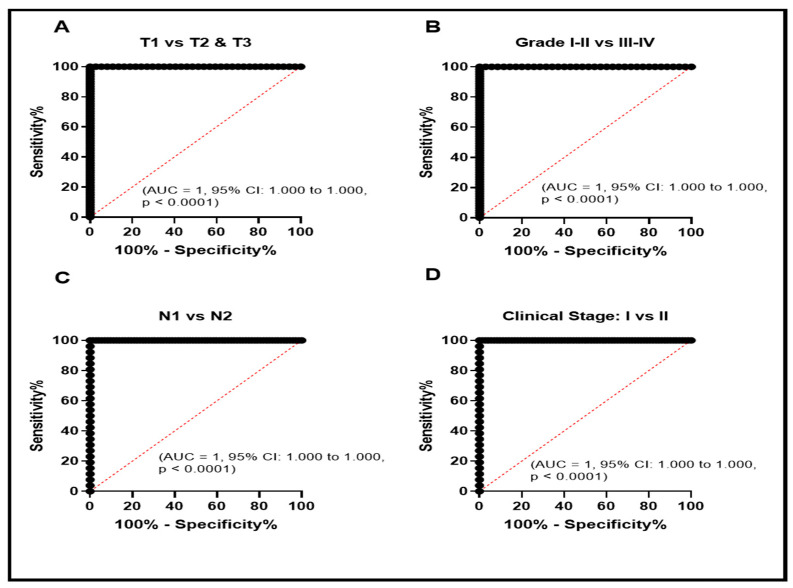
ROC curve evaluation of elevated GPX1-mRNA in patients with MPM with different clinical and pathological data to study its potential role as a diagnostic biomarker. ROC curve study for tumor size (T) (**A**), pathological grade (**B**), lymph node infiltration (N) (**C**), and TNM clinical stage (**D**).

To evaluate the diagnostic role of GPX1-mRNA in MPM, ROC curve was plotted between the GPX1-mRNA levels in the normal pleural tissues and MPM tissues. It revealed a high specificity to the diagnosis of MPM (Figure 1B). We plotted the ROC curve to evaluate the diagnostic significance of a high level of GPX1-mRNA in different clinical and pathological features. We observed that high GPX1-mRNA might be of clinical significance in the diagnosis of T1 patients/T2 + T3 (Figure 2A, AUC = 1.95%, CI: 1.000 to 1.000, *p* < 0.0001), grade 1 + 2/grade 3 + 4 (Figure 2B, AUC = 1.95%, CI: 1.000 to 1.000, *p* < 0.0001), N1/N2 lymph node infiltration (Figure 2C, AUC = 1.95%, CI: 1.000 to 1.000, *p* < 0.0001), and TNM clinical stage I/II (Figure 2D, AUC = 1.95%, CI: 1.000 to 1.000, *p* < 0.0001).

**Figure 3:**
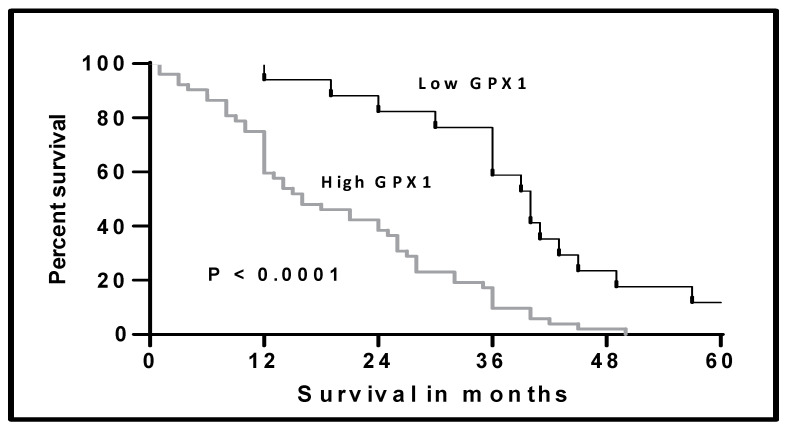
Kaplan–Meier curve of the overall survival in the patients with MPM according to the level of GPX1-mRNA in patients with MPM, Log-rank test = 17.59, *p* < 0.0001.

We studied the survival among our included patients according to the level of GPX1-mRNA by plotting the Kaplan–Meier curve (Figure 3). The median survival of the patients with low GPX1-mRNA was 40 months compared to 16 months for the patients with high levels. This difference in survival was statistically significant (Log-rank test 17.59, *p* < 0.0001).

## 4. Discussion

Mesothelioma is a fatal malignant tumor related to previous exposure to asbestos, originating from the surface of the pleura and presented in an advanced stage [1]. Early detection and diagnosis of this disease is the cornerstone in the management and the prognosis affected patients [16,17,18]. Up until now, there are no beneficial biomarkers for early diagnosis of pleural mesothelioma.

During the normal oxidative respiratory process, normal and malignant cells produce reactive oxygen species (ROS) that are eliminated by antioxidant enzymes. These ROS can damage DNA and other cellular organelles [6]. 

In malignant cells, there is an increase in the metabolic rate and disturbance in the mitochondrial function that results in increased ROS formation [19]. Accumulation of ROS resulted in malignant transformation, development, spread, and the emergence of resistant cells to cytotoxic drugs [20,21,22,23].

One of the members of the glutathione peroxidases (GPXs) is the GPX1 found in the cytoplasm and mitochondria. Many authors have reported that GPX1 can perform opposing functions in various cancers.

Elevated GPX1 production in squamous cell carcinoma of the oral cavity and the esophagus induced the tumor growth, spread, vascular infiltration, and resistance to Cisplatinum [12]. On the other side, increased GPX1 levels activated apoptosis in pancreatic carcinoma [24]. The role of GPX1 was not previously studied in MPM. In this research, we aimed to evaluate the function of GPX1 and its diagnostic and prognostic role in MPM.

In the current study, we observed that GPX1-mRNA levels were markedly elevated relative to the nearby normal pleural tissues. We noted that this elevation has a diagnostic specificity to differentiate MPM from normal tissues. Increased GPX1 expression was reported in the head and neck [25], gastric [26], and hepatic and prostatic malignancies [27]. On the other hand, low GPX1 expression was reported in thyroid cancer [28].

In our study, we noted that elevation of GPX1 was found in patients with adverse prognostic features such as old age, female gender, bad performance status, non-epithelioid tumors, undifferentiated tumors, large tumors, lymph node infiltration, unresectable tumors, advanced clinical stage, the presence of anemia, increased platelet and white blood cell counts, and increased serum LDH level.

Our results were also reported by Cheng et al. in renal cell carcinomas; they found that the increased levels of GPX1 were linked to lymph node metastases, advanced stage, and metastatic disease [29]. Lee et al. reported previously that elevated levels of GPX1 were related to adverse clinical features in oral squamous cell carcinomas [12]. On the contrary, in a previous study that included patients with gastric cancers, low expression of GPX1 was associated with aggressive tumors [30]. In head and neck squamous cell carcinomas, Dequanter et al. also reported the link between low GPX1 expression and advanced tumor stage [25].

To evaluate the prognostic significance of elevated GPX1 in MPM, we studied the survival of patients according to the GPX1-mRNA levels. The median overall survival was 16 months in patients with high GPX1 levels and 40 months in low GPX1 levels. Lee et al. reported in their series of patients with oral squamous cell carcinoma that high GPX1 levels were associated with short overall survival [12]. On the contrary, Ekoue et al. published that GPX1 expression levels have no impact on survival in patients with prostatic adenocarcinoma [31].

From previous studies and our study, we noted that GPX1 functions with different behaviors based on the tumor type. Besides, the molecular basis of GPX1 needs to be more explored.

## 5. Conclusions

In conclusion, GPX1-mRNA high expression was noted in MPM and was linked to adverse prognosis and poor survival. It can be used as a possible biomarker for diagnosis and prognosis in MPM.

## Figures and Tables

**Table 1:** The relation between GPX1-mRNA levels and different prognostic, clinical, and pathological features.

	GPX1 mRNA Level			
	No	Mean	SD	*p*
Age				
≤55 years >55 years	58 40	5.769 8.789	1.836 0.33	<0.0001 *
Sex				
Males Females	44 54	5.157 8.504	1.693 0.571	<0.0001 *
Pathology				
Epithelioid Non-Epithelioid	66 32	6.065 8.932	1.899 0.1485	<0.0001 *
Histopathological grade				
G1–G2 G3–G4	35 63	4.67 8.297	1.56 0.737	<0.0001 *
Tumor Resection				
Complete Partial Unresectable	37 49 12	4.79 8.17 9.06	1.60 0.68 0.03	<0.0001 **
T				
T1 T2 T3	72 26	6.282 8.993	1.956 0.075	<0.0001 *
N				
N1 N2	72 26	6.282 8.993	1.956 0.075	<0.0001 *
Stage				
I II	39 59	8.81 5.81	0.308 1.84	<0.0001 *
Hb%				
<14 gm >14 gm	56 42	5.69 8.75	1.819 0.366	<0.0001 *
Platelet Count				
Normal High	46 52	5.253 8.548	1.717 0.536	<0.0001 *
WBCs				
Normal High	57 41	5.729 8.77	1.827 0.3483	<0.0001 *
LDH				
Normal High	72 26	6.28 8.99	1.96 0.075	<0.0001 *
Performance status				
0–1 2	72 26	6.28 8.99	1.96 0.075	<0.0001 *

* Student’s t-test, ** one way analysis of variance (ANOVA).

## Data Availability

Data available on request due to privacy/ethical restrictions.
